# Careful Breakthrough Cancer Pain Treatment through Rapid-Onset Transmucosal Fentanyl Improves the Quality of Life in Cancer Patients: Results from the BEST Multicenter Study

**DOI:** 10.3390/jcm9041003

**Published:** 2020-04-02

**Authors:** Arturo Cuomo, Marco Cascella, Cira Antonietta Forte, Sabrina Bimonte, Gennaro Esposito, Stefano De Santis, Luigi Cavanna, Flavio Fusco, Mario Dauri, Silvia Natoli, Marco Maltoni, Alessandro Morabito, Rocco Domenico Mediati, Vito Lorusso, Sandro Barni, Giampiero Porzio, Sebastiano Mercadante, Anna Crispo

**Affiliations:** 1 Division of Anesthesia and Pain Medicine, Istituto Nazionale Tumori, IRCCS. Fondazione G. Pascale, 80131 Napoli, Italy; a.cuomo@istitutotumori.na.it (A.C.); c.forte@istitutotumori.na.it (C.A.F.); s.bimonte@istitutotumori.na.it (S.B.); gennaro.esposito@istitutotumori.na.it (G.E.); 2 Palliative Care and Oncologic Pain Service, S. Camillo-Forlanini Hospital, 00149 Rome, Italy; sdesantis@scamilloforlanini.rm.it; 3 Department of Oncology-Hematology, G. da Saliceto Hospital, 29121 Piacenza, Italy; l.cavanna@ausl.pc.it; 4 Palliative Home Care Unit, ASL 3, 16153 Genova, Italy; flavio.fusco@asl3.liguria.it; 5 Department of Anesthesiology and Resuscitation, II University of Rome, Tor Vergata, 00133 Roma, Italy; mario.dauri@uniroma2.it (M.D.); silvia.natoli@uniroma2.it (S.N.); 6 Palliative Care Unit, Istituto Scientifico Romagnolo per lo Studio e la Cura dei Tumori (IRST) IRCCS, 47014 Meldola, Italy; ma.maltoni@ausl.fo.it; 7 Medical Oncology Unit, Istituto Nazionale Tumori, IRCCS. Fondazione G. Pascale, 80131 Napoli, Italy; a.morabito@istitutotumori.na.it; 8 Palliative Care and Pain Therapy Unit, University Hospital Careggi, 50134 Firenze, Italy; mediatir@aou-careggi.toscana.it; 9 UO Oncologia Medica, Istituto Tumori “G Paolo II”, 70124 Bari, Italy; vitolorusso@me.com; 10 Oncology Unit, ASST Bergamo Ovest, 24047 Treviglio, Italy; sandro_barni@asst-bgovest.it; 11 Medical Oncology Unit, San Salvatore Hospital, University of L’Aquila, 67100 L’Aquila, Italy; terapiesupportoaq@interfree.it; 12 Main regional center for Pain Relief & Supportive Care, La Maddalena Cancer Center, 90146 Palermo, Italy; terapiadeldolore@lamaddalenanet.it; 13 Epidemiology and Biostatistics Unit, Istituto Nazionale Tumori, IRCCS. Fondazione G. Pascale, 80131 Napoli, Italy; a.crispo@istitutotumori.na.it

**Keywords:** breakthrough cancer pain, cancer-associated pain, cancer, health-related quality of life, sleep disorders, transmucosal fentanyl

## Abstract

Objectives: To explore the effect of breakthrough cancer pain (BTcP) treatment on quality of sleep and other aspects of the health-related quality of life (HRQoL) in patients with cancer pain. Methods: In an observational, multicenter, cohort study, cancer patients from palliative care units, oncology departments, and pain clinics and affected by BTcP were included. Enrolled patients were assessed at the four visits: T0 (baseline), T7, T14, and T28. Stable chronic background pain (numeric rating scale, NRS ≤ 4) during the whole study period was mandatory. BTcP was treated through transmucosal fentanyl. Three questionnaires were used to measure the HRQoL: EORTC QLQ-C15-PAL, Pittsburgh Sleep Quality Index (PSQI), and the Edmonton Symptom Assessment System (ESAS). RESULTS: In 154 patients, the HRQoL showed a significant improvement for all physical and emotional characteristics in the EORTC QLQ-C15-PAL, except for nausea and vomiting (linear *p*-value = 0.1) and dyspnea (Linear *p*-value = 0.05). The ESAS and PSQI questionnaires confirmed these positive results (*p* < 0.0001 and *p* = 0.002, respectively). Conclusions: This prospective investigation by an Italian expert group, has confirmed that careful management of BTcP induces a paramount improvement on the HRQoL. Because in cancer patients there is a high prevalence of BTcP and this severe acute pain has deleterious consequences, this information can have an important clinical significance.

## 1. Introduction

Pain is one of the most frequent symptoms in cancer patients as it occurs in 20–30% of cases during the initial stages and in up to 75% of patients in advanced disease. The prevalence of cancer pain at any stage of the disease is over 50%. Concerning pain intensity, moderate to severe pain can affect up to 40% of all patients. Furthermore, this symptom strongly affects the patient’s health-related quality of life (HRQoL) and daily activities throughout the cancer disease [1]. As a consequence, in this clinical setting, it is mandatory to relieve pain and other symptoms and to improve the HRQoL at any stage of the disease [2]. Despite the clear validity of this statement, the availability of several guidelines on the topic [3], and evidence that effective pain-relieving strategies can improve HRQoL and adherence to anticancer treatment [4], cancer-pain management remains an impressive challenge in medicine. 

Assessment and management of cancer pain become particularly critical when the patient faces temporary exacerbations of pain despite adequate control with opioids. This phenomenon, termed as breakthrough cancer pain (BTcP), is a very frequent condition as approximately 70% of patients suffering from chronic pain of oncological nature report episodes of BTcP [5]. Typical BTcP episodes are of short duration (15–30 min/episode), moderate to severe intensity and with rapid onset (maximum peak between 3–15 min). Our recent investigations conducted on a large sample of patients (*n* = 4016), dissected the features of this painful phenomenon and demonstrated that in 86% of patients the occurrence of BTcP induced a marked interference with their daily activities [6]. Moreover, several pieces of evidence demonstrated that BTcP is associated with negative outcomes for both patients and healthcare providers [7]. Thus, during the IOPS study, we realized that the impact of pain therapy on some aspects of the HRQoL required further investigation. Because previous studies showed that cancer-related poor sleep quality can lead to a worsening in psychological, and physical functions [8], the next step was to evaluate whether an effective background analgesia control combined with proper management of the BTcP could lead to an improvement of the sleep quality and the overall HRQoL. 

The present observational study—indicated as breakthrough cancer pain evaluation study (BEST-Study)—aimed at assessing the impact of BTcP management on HRQoL, sleep and daily activities in patients from different clinical settings and with adequately controlled background pain through strong opioids. 

## 2. Materials and Methods

### 2.1. Study Population and Design

This clinical study was conducting by following the Declaration of Helsinki’s ethical principles. Individuals with a diagnosis of any type of solid cancer and affected by chronic pain effectively controlled—numeric rating scale (NRS) ≤4—by around-the-clock (ATC) opioid maintenance therapy through ≥ 60 mg of oral morphine equivalent daily doses (OMEDD) on the day of enrollment, were included in this national multicenter prospective cohort study, from March 2015 to August 2017. Approval from the Institutional Medical Ethical Committee (protocol 32/14 OSS) of the Istituto Nazionale Tumori-Fondazione Pascale, Naples was obtained, and patients signed informed consent before enrolling in the study. The Strengthening Reporting of Observational Studies in Epidemiology (STROBE) guidelines were followed [9].

**Figure 1:**
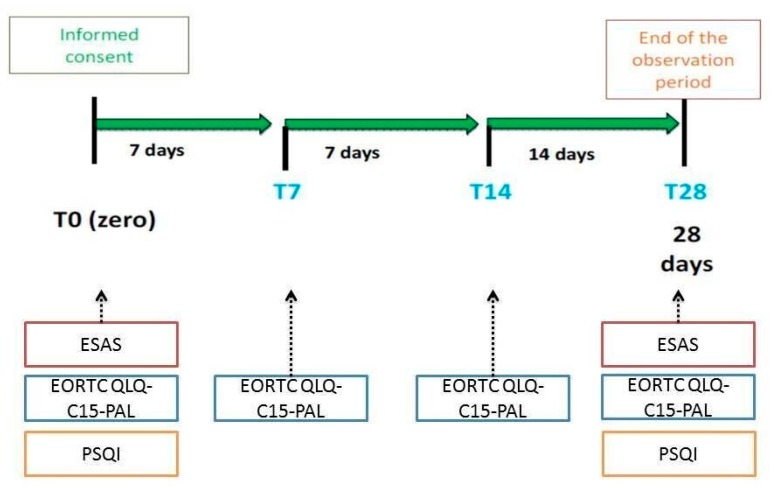
Study design. Legend: EORTC QLQ-C15-PAL, European Organization of Research and Treatment Quality of Life Questionnaire-Cancer 15; PSQI, Pittsburgh Sleep Quality Index; ESAS, Edmonton Symptom Assessment System.

Patients’ eligibilities were evaluated by an oncologist or pain specialist or palliative care specialist and in-hospital patients, outpatients, and those in home-care settings, were included. Each participating patient was observed for a maximum of 28 days. During the study, patients underwent to four consultations starting from baseline examination (T0) for enrolment and data collection. In particular, in this initial step demographic characteristics, settings, tumor type, background pain characterization (the type of pain and pain intensity), and performance status through the Eastern Cooperative Oncology Group (ECOG) 0–5 scale, were collected. Each participating patient was then evaluated after 7 days (T7), 14 days (T14), and 28 days from baseline (T28), (Figure 1).

Data were collected from the patient’s medical records and summarized in e-CRF (electronic Case Report Form). The definition of BTcP was: a transitory pain exacerbation of moderate to severe intensity that occurs spontaneously or predictably [10], well distinguished from a stable background pain (NRS ≤ 4) [11]. BTcP treatment was carried out through one of the available rapid-onset transmucosal formulations. Inclusion and exclusion criteria are summarized in Table 1.

### 2.2. Study Endpoints and HRQoL Assessments

The primary objective of the study was to define:

- The impact on BTcP treatment through transmucosal fentanyl on HRQoL, sleep quality and daily activities in patients with background pain adequately controlled by ATC opioids as maintenance therapy.

The HR quality of life (QoL) was measured by the European Organization of Research and Treatment Quality of Life Questionnaire-Cancer 15 (EORTC QLQ-C15-PAL) [12] and the Edmonton Symptom Assessment System (ESAS) [13] whereas sleep quality was assessed by the Pittsburgh Sleep Quality Index (PSQI) [14]. EORTC QLQ-C15-PAL was administered at the four visits: T0 (baseline), T7, T14, and T28, whereas ESAS and PSQI were adopted at the baseline (T0) and the end of the observation period (T28). 

The secondary objective was to evaluate the:

- Baseline background pain characteristics;
- BTcP features including type (spontaneous, incident), and the number of episodes. Differences according to visits (T0, T7, T14, T28) on number of BTcP episodes were evaluated;
- BTcP management in terms of median time to pain relief.

Any serious adverse event related to background pain therapy and for BTcP management was reported.

### 2.3. Statistical Analyses

#### 2.3.1. General Linear Model

Categorical variables were presented by frequencies and percentages. The general linear model (GLM) for repeated measures analysis was used. This statistical approach is mathematically identical to multiple regression analysis and it is particularly useful for the correlation of multiple qualitative and quantitative variables. According to this strategy, the mean-scores for HRQoL parameters in T0, T7, T14, and T28 were evaluated: the linear *p*-value indicates the statistical trend for the four measures, while the square *p*-value shows the statistical change from the last visit to the baseline (T28 vs. T0). 

Results were only based on non-missing data (i.e., no replacement of missing observations was applied), unless specified otherwise; in particular, the number of patients that completed each visit was taken into account: T0 = 154 pts; T7 = 136 pts; T14 = 124 pts and T28 = 100 pts. 

The Bonferroni’s method can be used to compare different groups at the baseline. It provides a pairwise comparison of the means, investigates on the relationship between variables, or examines one or more endpoints in clinical trials. To obtain effect size, partial eta-squared (η2p) was calculated. Estimates of effect size give a partial eta squared (η2p) value for each effect and each parameter considered. The partial eta-squared statistic describes the proportion of the total variability attributable to a factor (small = 0.01, medium = 0.06, and large = 0.14). 

Statistical analyses were performed using IBM^®^ SPSS^®^ Statistics, version 25 (IBM Corp., Armonk, NY, USA). All statistical tests were two-sided, and *p* values of less than 0.05 were considered significant.

#### 2.3.2. Quality of Life Analysis 

We used a mathematical approach that could allow us to obtain the greatest amount of information despite the reduced sample size. In particular, since each score represented a continuous quantity, to make the variable discrete, we applied a statistical approach proposed by Osaba et al. [15] which allows establishing the improvement or the worsening, setting in advance what the variation of the score must be (e.g., of 10 points). In short, each patient represents his control. According to this method, the best HRQoL response from baseline for each domain or symptom was estimated as a change of the score of at least 10 points from baseline to be clinically relevant. Subjects were intended as improved if they achieved a score ≥10 points than baseline anytime, and were judged worsened with a score ≥10 points lower than baseline without having improved at any time; those with scores fluctuating less than 10 points from baseline were intended stable. A Chi-square test was used to test statistical significance.

An adjusted logistic regression analysis was performed to assess the association between the best response of HRQoL questionnaires (EORTC; PSQI and ESAS) and some covariates. Odds ratios (ORs) and 95% confidence intervals (95% CI) were estimated. 

## 3. Results 

Table 2 summarizes demographic and clinical data for the population under study. Among the eligible patients, 154 who referred to control cancer pain by ATC opioid therapy maintenance and suffering from BTcP were included in the analysis. The mean age was 63 ± 11 years, 56% were men and 44% women. A total of 92 patients were treated in an oncological department, 36 by pain therapists, and 26 in a palliative care setting. Background pain was nociceptive in 37% of patients and mixed (nociceptive plus neuropathic) in the remaining 63%.

### 3.1. Quality of Life

**Figure 2:**
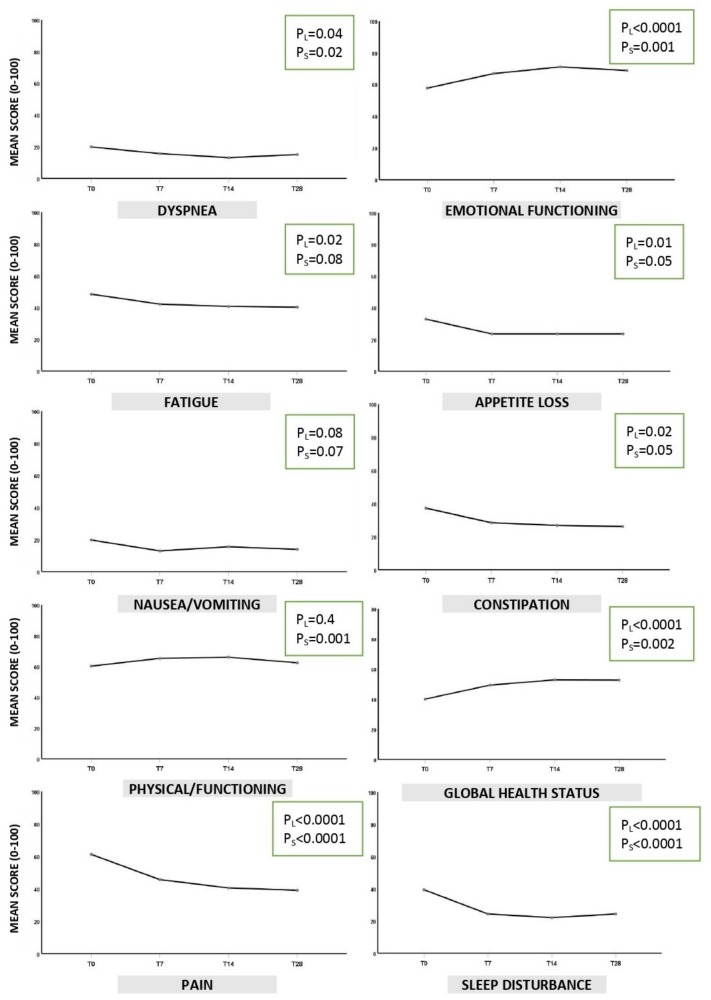
EORTC QLQ-C15-PAL results. Legend: P_L_ = Linear *p*-value indicates the statistical trend for the 4 visits; P_S_ = square *p*-value indicates the statistical change from T28 to T0. *p* < 0.05 was considered statistically significant for both.

The HRQoL analysis through GLM showed a significant improvement for all physical and emotional characteristics in the EORTC QLQ-C15-PAL, except for nausea and vomiting (linear *p*-value = 0.1), and dyspnea (linear *p*-value = 0.05). Moreover, there was a significant improvement in the global health status at the end of the study (*p* = 0.002) (Figure 2).

PSQI and ESAS questionnaires confirmed a significant improvement in the quality of sleep (PSQI), and in all domains of the HRQoL including psychological outcomes (ESAS), after 28 days of observation (*p* = 0.002, and *p* < 0.0001 respectively) (Table 3). Concerning effect size of GLM analysis, Table 3 compares each follow-up (T7, T14, T28) at baseline for each tool administered. Significant results with largest effect size were observed for Global health status/QoL, pain and ESAS (η2p = 0.14; 0.26 and 0.14, respectively).

Consistent results were found when QoL best response was calculated (Table 4A). The improved were 55% for the Global health status of the EORTC tool; about 40% for PSQI, and 62% for ESAS. Interestingly, the female gender was associated with an improvement in global health status (68%) (Table 4B). On the other hand, the lowest number of improved were observed for nausea/vomiting, and dyspnea (27% and 29%, respectively), confirming the significant results from GLM analysis (Figure 2 and Table 3). 

Subsequently, the improved were compared with worse response to assess variables that contributed to explain this difference for the three questionnaires adopted (EORTC QLQ-C15-PAL, PSQI, and ESAS). Some covariates were also studied. Table 5 shows the results of the adjusted logistic regression analysis. A significant improvement for the global health status (EORTC) was found for female patients (OR = 0.17 95% CI 0.04–0.68), and the setting of patients treated by pain therapists compared with those managed by oncologists (OR = 0.11 95% CI 0.02–0.58). This latter significant result was confirmed either for the sleep quality (PSQI) or ESAS questionnaires (OR = 0.15 95% CI 0.03–0.77; OR = 0.27 95% CI 0.09–0.85, respectively). Furthermore, a significant association was found with the type of BTcP. In particular, the presence of mixed pain was associated with a better response in the global health status (OR = 0.17 95% CI 0.05–0.67) compared with the nociceptive pain alone (*p*= 0.01). Finally, data from the PSQI questionnaire indicated that the older age (>70) is significantly associated with worse sleep quality (OR = 8.46 95% CI 2.46–29.11). 

### 3.2. Breakthrough Cancer Pain

**Figure 3:**
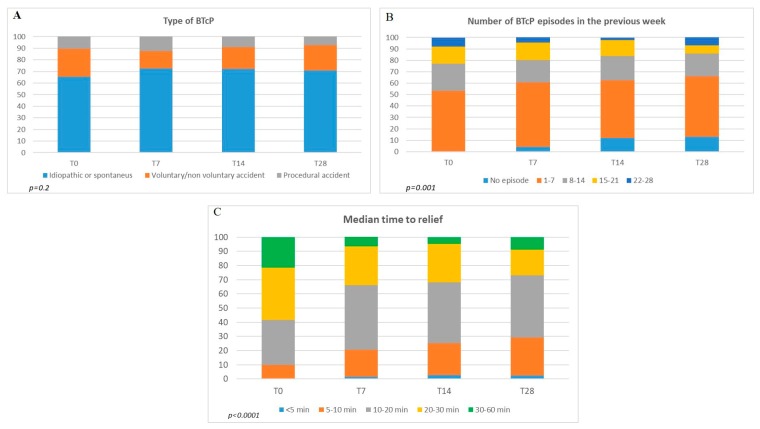
Breakthrough cancer pain (BTcP) results. (**A**) Features, (**B**) trend, and (**C**) response to therapy.

Figure 3 illustrates the features of BTcP in patients who completed the study (real percentage). At baseline, BTcP was mainly spontaneous/idiopathic (70% of all registered cases), the incident type (voluntary or non-voluntary) occurred in 21% of cases, while in the remaining 9% a procedural BTcP was found. In the course of the study, this distribution did not change (not significant, *p* = 0.2) (Figure 3A). At T0, the BTcP number of episodes (1 to 7; 8 to 14; 15 to 21; 22 to 28), showed that 1–7 BTcP episodes per week occurred in approximately 53% of all cases and a high number of events (22 to 28) were reported by 7.8% of patients. During the observation times, the number of patients with many episodes of BTcP (22 to 28) decreased (T7 4.4%; T14 2.4%) but returned to increase at the end of the study (7%). We also found that the amount of patients who were free from BTcP increased significantly during the study (from 0 to 13%) (*p* < 0.001). The reduction was also significant for the clusters 8-14 and 15-21 events. In particular, 15-21 episodes at baseline were reported for about 15% of patient to decrease to 7% at T28 (*p* < 0.001) (Figure 3B). Concerning the medium time to relief, while at T0 it was 30–60 minutes in about 20% of patients, at the end of the study the percentage of patients who responded in long times dropped to 7% (*p* < 0.0001 ). Furthermore, the number of patients who responded to treatment in 20–30 minutes also dropped significantly from about 29.2% to 15% (*p* < 0.0001). On the contrary, the response increased in ultra-short (<5 min) and short (5–10, 10–20) times. In addition, in these ranges, the variations were significant (*p* < 0.0001) (Figure 3C).

## 4. Study Limitations

The major limitation of the study seems to be the poor consistency of the sample size. For instance, the small size simple and the lack of a complete multivariate analysis, associated with the rapid-onset opioids (ROO) pharmacokinetics features (i.e., time limited activity) made it difficult to establish how the therapeutic variable was related—in a cause-effect relationship—with the type of response. However, thanks to the statistical approach adopted which combined the multivariate analysis with the careful interpretation of the changes in the HRQoL scores, we were able to measure the change in the four observation times for each patient enrolled, estimating, in turn, the trend. In other words, an ’outcome’ variable was established making the analysis independent from the sample size. In addition, adjusted multivariate analysis was also applied to the three questionnaires. 

Another limitation is the lack of data on the type of treatment of background pain (e.g., opioids and their doses, adjuvants, other pharmacological and non-pharmacological therapies). As a characterization of therapy was not the aim of the study, we considered all patients with OMEDD of ≥ 60 mg and well-controlled pain to be enrolled. 

Finally, the lack of the control group due to the study design did not allow for a precise analysis of different dose response patterns. We hope that the data from this observational investigation can be used to design controlled studies.

## 5. Discussion

This study explored the effect of BTcP treatment on several aspects of the HRQoL in patients with cancer pain. The HRQoL was investigated through a battery of tools capable of evaluating the largest number of domains. General data were investigated by the EORTC QLQ-C15-PAL, the PSQI questionnaire served to evaluate sleep quality, and the ESAS one helped to explore general domains and psychological aspects of cancer pain including anxiety and depression.

In regards to BTcP management, we have chosen to treat BTcP through rapid-onset opioids (ROOs), drugs specifically indicated for this purpose. ROOs, indeed, have a pharmacodynamics that mirror the sudden start and brief duration of the pain event [3]. However, because the lack of comparative data, we have not indicated the type of formulation to be used. Furthermore, despite there is a debate on the starting dose of ROOs, i.e., if a titration dose is needed or these drugs must be administered in proportional to the regimen of opioid for background pain treatment [16], in this study we have adopted the ROOs administration strategy based on the following the provisions of the relative summary of product characteristics, and in absence of specific contraindications.

Although the HRQoL investigated through the EORTC QLQ-C15-PAL instrument showed a significant amelioration in the global health status at the end of the study and a significant improvement for all physical and emotional descriptors of the questionnaire, except for nausea and vomiting, and dyspnea. Concerning dyspnea, it is a symptom of advanced disease, whereas nausea and vomiting are mainly related to opioid therapy but can be also related to the cancer disease itself, and anticancer therapies. Having observed an improvement for constipation and not for nausea and vomiting, perhaps may indicate that during opioid therapy more attention is paid to constipation than to other adverse effects that only apparently have a lower impact on HRQoL. 

Concerning quality of sleep, compared to the baseline we observed a significant improvement (*p* < 0.0001) at the end of the study. Nevertheless, the adjusted logistic regression analysis demonstrated that the elderly patients (>70 years) had a risk 8.5 fold higher than those observed in patients less than 60 years of age. These findings are not surprising as a recent cross-sectional multicenter study has highlighted that around 80% of cancer patients experienced poor sleep quality, and this issue mainly concerns older patients [17]. The clinical implication is that sleep quality must be better assessed and greater efforts must be made to ensure adequate treatment of sleep disturbances in these patients.

The ESAS questionnaire also showed a significant improvement between the end and the beginning of the study (*p* < 0.0001). However, the regression analysis failed to find a correlation between these positive results and age-related factors, gender, and type of underlying pain. Whatever the underlying correlation, we can assume that the treatment of cancer pain induces an important improvement of multiple patient’s psychological aspects [18]. 

Further significant data came from the regression analysis. Female gender, for example, was associated with an improvement in global health status (*p* < 0.01). Several pieces of evidence suggested that different pain thresholds may exist between men and women. Physiologic factors such as ovarian hormone and increased serotonergic activity, and psychological dynamics (e.g., increased anxiety in women) have been called into question to explain this phenomenon which, in turn, must be better explained [19]. However, it seems to be difficult to explain a better therapeutic result in terms of pain relief in one sex compared to the other. Preclinical research in rodents showed different concentration of opioid receptors between male and female animals. Furthermore, morphine seems to induce more potent effects in female than in male [20]. Again, it was proved that female rats require almost twice as much morphine as males for obtaining comparable analgesic effects [21]. Probably, sex differences in opioids response must be better elucidated. Furthermore, the gender-related improvement concerned not only the symptom pain but involved other aspects of HRQoL, except for sleep (PSQI) and the domains of the ESAS tool.

Of note, the main finding that is found in all questionnaires is the paramount role of pain therapists as there was significant improvement in the setting of patients treated by these clinicians. Pain therapists are perhaps more likely to perform pain management through tailored therapies and by coordinating multidisciplinary strategies [22]. The improvement also affected patients treated in palliative care, although the result was not significant. 

With regards to background pain, the best results have been achieved in patients with mixed pain, although only in the EORTC questionnaire. The data can be explained with probable greater attention towards forms of pain that represent a challenge for those who must treat chronic pain.

The analysis of BTcP and its trend during the investigation period showed interesting data. Our study suggested that adequate BTcP management can probably reverberate on the characteristics of BTcP by reducing the number of episodes. Of note, there was also an improved response to therapy. The phenomenon, however, requires further investigation as multiple factors such as type and course of cancer disease, clinical setting, and background pain therapy can influence the incidence and characteristics of BTcP. Furthermore, this unpredictable severe acute pain represents a heterogeneous condition and includes spontaneous forms and those induced by voluntary, or involuntary movements. Therefore, especially incident pain benefits from better therapy. In a longitudinal study, carried out on in-hospital patients, the incidence changed over time, falling from 87% to 32% after six months from the first detection. The greatest decrease in BTcP occurrence concerned the incident subtype [23]. Moreover, Mercadante et al. [24] found in home-care patients, that the prevalence of BTcP decreased after one month, possibly due to a progressive reduction in physical activity or as a consequence of a better background pain control. Thus, we have planned to carry out another multicenter research and a retrospective analysis on a large database, for evaluating potential associations between BTcP and several clinical variables. 

## 6. Conclusions

BTcP treatment remains a great challenge in medicine. It requires, indeed, individualized treatment through the involvement of a multidisciplinary team. Despite the limitations, results from this multicenter, observational cohort study conducted by an Italian expert group, has confirmed that accurate management of BTcP from the baseline may improve several aspects of the HRQoL. Because of in cancer patients there is a high prevalence of BTcP and this severe acute pain has deleterious clinical consequences, this information can have an important clinical significance. In this population, more attention should be given to the treatment of sleep disturbances and the management of nausea and vomiting. Further studies are needed to verify the fascinating hypothesis that adequate BTcP treatment can probably reduce the number of episodes, also improving its response to therapy, and in turn, the HRQoL of these fragile individuals. 

## Figures and Tables

**Table 1:** Inclusion and exclusion criteria.

Inclusion Criteria	Exclusion Criteria
Adult male and female patients (≥18 years) of any ethnic origin with solid malignancy (any stage) Stable chronic background pain: baseline NRS ≤4 for more than 12 h a day in the previous week through ATC opioid therapy Patients who report suffering from BTcP crisis: 1 to 4 times a day in at least 1 of the 3 days before the start of the study Prescription of transmucosal fentanyl (any formulation) for the treatment of BTcP in doses effective according to the best supportive therapy and in accordance with the provisions of the relative SmPC, in absence of specific contraindications Basic therapy of chronic background pain through OMEDD of ≥60 mg Patient’s ability to understand and sign the informed consent Life expectancy of at least 30 days ECOG Performance Status 0 to 3	Intense episodic pain of a non-oncological nature Intense no-BTcP * ECOG Performance Status 4 Altered patient’s state of consciousness and/or inability to fill in the evaluation questionnaires Participation in an intervention clinical study Pregnancy or breastfeeding Contraindications to the use of opioids BTcP treatment already in place Patients with previous or current history of neurological/psychiatric disorder and/or any substance abuse (or dependence) ^ Any medical condition or situation complicating the collection of study data, as determined by the Investigator

Notes: * End-dose pain or pain during titration of the opioid dose; ^ patients treated with antidepressants have been not excluded. Abbreviations: NRS, Numeric Rating Scale; ATC, around-the-clock; OMEDD, oral morphine equivalent daily doses; BTcP, breakthrough cancer pain; SmPC, summary of product characteristics; ECOG, Eastern Cooperative Oncology Group.

**Table 2:** Baseline demographic and clinical data (*n* = 154).

Sex	*N* (%)
Male	86 (55.8)
Female	68 (44.2)
Age (mean ± SD)	63.5 ± 11.2
Tumor type, *n* (%)	
Lung	51 (33.1)
Breast/Gynecological	12 (7.8)
Gastrointestinal	20 (13.0)
Others #	71 (46.1)
Pain management	
Oncology	92 (59.7)
Pain therapy	36 (23.4)
Palliative care	26 (16.9)
Settings	
Hospital patients	88 (57.1)
Outpatients	61 (39.6)
Home-care settings	5 (3.2)
Background pain	
Nociceptive	62 (37.1)
Mixed pain	105 (62.9)

Legend: # sarcomas, melanomas and other skin cancers, bone tumors, urological tumors.

**Table 3:** General linear model analyses: mean score of quality of life at baseline until last follow-up.

	Baseline Mean (I) ± SD	Follow-Up			Partial Square Eta (η^2^_p_) **
		T7	T14	T28	
		Mean (J) ± SD	Mean (J) ± SD	Mean (J) ± SD	
**EORTC**					
**Global health status/QoL**	40.06 ± 20.4	49.36 ± 18.7	52.88 ± 22.2	52.72 ± 23.7	
Mean differences (I-J) (*p*-value) *		−9.30 (<0.0001)	−12.82 (<0.0001)	−12.66 (<0.0001)	0.14
**Physical functioning**	60.25 ± 28.3	65.27 ± 24.6	66.03 ± 26.9	62.4 ± 30.1	
Mean differences (I-J) (*p*-value) *		−5.02 (0.06)	−5.76 (0.05)	−2.14 (0.9)	0.03
**Emotional functioning**	57.53 ± 25.2	66.66 ± 24.1	70.83 ± 25.1	68.58 ± 26.4	
Mean differences (I-J) (*p*-value) *		−9.13 (0.001)	−13.3 (<0.0001)	−11.06 (0.001)	0.10
**Fatigue**	48.39 ± 28.3	42.15 ± 25.7	40.7 ± 26.7	40.22 ± 28.3	
Mean differences (I-J) (*p*-value) *		6.25 (0.1)	7.69 (0.02)	8.17 (0.08)	0.04
**Nausea/vomiting**	19.74 ± 25.3	12.94 ± 19.9	15.53 ± 22.7	13.91 ± 22.1	
Mean differences (I-J) (*p*-value) *		6.79 (0.01)	4.20 (0.4)	5.82 (0.2)	0.03
**Pain**	61.21 ± 22.8	45.67 ± 22.5	40.54 ± 25.4	39.10 ± 22.5	
Mean differences (I-J) (*p*-value) *		15.54 (<0.0001)	20.67 (<0.0001)	22.11 (<0.0001)	0.26
**Dyspnea**	19.87 ± 25.2	15.7 ± 21.3	13.14 ± 22.5	15.1 ± 23.6	
Mean differences (I-J) (*p*-value) *		4.17 (0.1)	6.73 (0.01)	4.80 (0.4)	0.03
**Sleeping disturbance**	39.4 ± 35.9	24.35 ± 27.2	22.11 ± 27.3	24.35 ± 28.3	
Mean differences (I-J) (*p*-value) *		15.06 (<0.0001)	17.3 (<0.0001)	15.06 (<0.0001)	0.13
**Appetite loss**	33.01 ± 31.6	23.71 ± 28.1	23.71 ± 30.3	23.71 ± 29.9	
Mean differences (I-J) (*p*-value) *		9.29 (0.008)	9.29 (0.03)	9.29 (0.04)	0.05
**Constipation**	37.22 ± 32.7	28.48 ± 27.7	26.89 ± 27.2	26.21 ± 29.01	
Mean differences (I-J) (*p*-value) *		8.74 (0.002)	2.9 (0.003)	11.0 (0.008)	
**PSQI Sleep disorders**	9.54 ± 4.3	-	-	8.29 ± 4.6	
Mean differences (I-J) (*p*-value) *				1.25 (0.001)	0.10
**ESAS**	29.95 ± 16.1	-	-	23.1 ± 16.9	
Mean differences (I-J) (*p*-value) *				6.86 (<0.0001)	0.14

Notes: * Bonferroni’s method (*p*-value); ** Partial eta-squared (η^2^_p_) small = 0.01, medium = 0.06, and large = 0.14. Abbreviations: EORTC QLQ-C15-PAL, European Organization of Research and Treatment Quality of Life Questionnaire-Cancer 15; PSQI, Pittsburgh Sleep Quality Index; ESAS, Edmonton Symptom Assessment System.

**Table 4:** Best quality of life response: overall data (**A**) and gender stratification (**B**).

Overall Response							Gender Stratification					
SCALE/ITEM	RESULT TREND ^											
	Improved		Stable		Worsened		Improved		Stable		Worsened	
	*n*	(%)	*n*	(%)	*n*	(%)	M *n (%)*	F *n (%)*	M *n (%)*	F *n (%)*	M *n (%)*	F *n (%)*
EORTC												
Global health status	55	(55)	27	(27)	18	(18)	28 (47)	27 (68)	17 (28)	10 (25)	15 (25)	3 (7)
Physical function.	39	(39)	26	(26)	35	(35)	22 (37)	17 (43)	16 (26)	10 (25)	22 (37)	13 (32)
Emotional function.	54	(54)	27	(27)	19	(19)	31 (52)	23 (58)	17 (28)	10 (25)	12 (20)	7 (17)
Fatigue	53	(53)	19	(19)	28	(28)	30 (50)	23 (58)	13 (22)	6 (15)	17 (28)	11 (27)
Nausea/vomiting	27	(27)	59	(59)	14	(14)	17 (28)	10 (25)	34 (57)	25 (63)	9 (15)	5 (12)
Pain	61	(61)	28	(28)	11	(11)	31 (52)	30 (75)	21 (35)	7 (18)	8 (13)	3 (7)
Dyspnea	29	(29)	60	(60)	11	(11)	23 (38)	6 (15)	32 (54)	28 (70)	5 (8)	6 (15)
Sleep disturbance	45	(45)	40	(40)	15	(15)	27 (45)	18 (45)	23 (38)	17 (43)	10 (17)	5 (12)
Appetite loss	38	(38)	42	(42)	20	(20)	20 (33)	18 (45)	26 (44)	16 (40)	14 (23)	6 (15)
Constipation	36	(36)	45	(45)	19	(19)	22 (37)	14 (35)	24 (40)	21 (53)	14 (23)	5 (12)
PSQI Sleep disorders	39	(40)	42	(43)	16	(17)	20 (34)	19 (50)	29 (49)	13 (34)	10 (17)	6 (16)
ESAS	62	(63)	6	(6)	31	(31)	37 (62)	25 (64)	4 (6)	2 (5)	19 (32)	12 (31)

Notes: ^ Improved=score ≥10 points were better than baseline anytime; worsened = score ≥10 points lower than baseline without having improved at any time; stable = score changes ≤ 10 points from baseline. Abbreviations: EORTC QLQ-C15-PAL, European Organization of Research and Treatment Quality of Life Questionnaire-Cancer 15; PSQI, Pittsburgh Sleep Quality Index; ESAS, Edmonton Symptom Assessment System.

**Table 5:** Adjusted logistic regression analysis on improved^ patients.

	Global health status *	PSQI	ESAS
	*p-*Value (OR 95% CI)	*p-*Value (OR 95% CI)	*p-*Value (OR 95% CI)
**Sex**	0.01	0.3	0.9
Male	1 †		
Female	0.17 (0.04–0.68)		
**Age**	0.7	0.002	0.4
<60		1 †	
61–70		1.68 (0.51–5.53)	
>70		8.46 (2.46–29.11)	
**Department**	0.03	0.07	0.02
Oncology	1 †	1 †	1 †
Pain therapy	0.11 (0.02–0.58)	0.15 (0.03–0.77)	0.27 (0.09–0.85)
Palliative care	0.53 (0.13–2.11)	0.81 (0.23–2.74)	0.46 (0.15–1.36)
**Type of Pain**	0.01	0.1	0.2
Nociceptive	1 †		
Neurop. /Nocic.	0.17 (0.05–0.67)		

Notes: ^ Score ≥ 10 points better than baseline anytime; * evaluated by the European Organization of Research and Treatment Quality of Life Questionnaire-Cancer 15 (EORTC QLQ-C15-PAL); † logistic regression adjusted for terms of sex, age. Abbreviations: PSQI, Pittsburgh Sleep Quality Index; ESAS, Edmonton Symptom Assessment System.
